# ITGB4 is a novel prognostic factor in colon cancer

**DOI:** 10.7150/jca.29269

**Published:** 2019-08-28

**Authors:** Meng Li, Xia Jiang, Guiqi Wang, Congjie Zhai, Ying Liu, Hongyan Li, Yan Zhang, Weifang Yu, Zengren Zhao

**Affiliations:** 1 Department of General Surgery, The First Hospital of Hebei Medical University, No. 89 Donggang Road, Shijiazhuang, Hebei, China; 2 The First Department of Colorectal Surgery, The Third Hospital of Hebei Medical University, No. 139 Ziqiang Road, Shijiazhuang, Hebei, China; 3 Department of Endoscopy Center, The First Hospital of Hebei Medical University, Donggang Road No. 89, Shijiazhuang, Hebei, China

**Keywords:** ITGB4, Expression, Prognosis, Colon cancer

## Abstract

Integrin β4 (ITGB4) has been reported to be involved in carcinomas. Currently, ITGB4 has been characterized in colon cancer, however, its clinical significance is not very clear. In the present study, we utilized the large public datasets from NCBI Gene Expression Omnibus (GEO) and The Cancer Genome Atlas (TCGA) databases and collected clinical samples in our center to investigate the transcriptional expressions of ITGB4 in colon cancer, and then explored the associations of ITGB4 with clinicopathological features and overall survival. The statistical analyses suggested that ITGB4 mRNA expressions were up-regulated significantly in colon cancer. High ITGB4 expression was observed to be associated with elder onset age, proximal tumor location, and high microsatellite instability (MSH) status. Further, Kaplan-Meier curves and univariate analysis demonstrated high ITGB4 expression was significantly associated with unfavorable overall survival in colon cancer (HR=1.292, 95%CI=1.084-1.540, P=0.004). And significant association was also found after adjusting the confounding factors including age, gender, and stage (adjusted HR=1.254, 95%CI=1.050-1.497, P=0.012). The annotation of ITGB4 co-expressed genes suggested the pathways including cell growth, positive regulation of cell migration, and apoptotic signaling might be involved in the potential mechanisms of ITGB4 in colon cancer development. The molecular regulation mechanism of ITGB4 ectopic expression in colon cancer was also explored and the results indicated that ITGB4 might be up-regulated by the transcription factor FOSL1 (FOS like 1, AP-1 Transcription Factor Subunit) and its promoter hypomethylation. Our results revealed that ITGB4 might be a therapeutic target and prognosis marker for individual therapy of colon cancer.

## Introduction

Colorectal cancers (CRC) occur in the colon (colon cancer) or rectum (rectal cancer) and are the fourth leading cause of cancer-related deaths around the world 1. Surgery and/or chemotherapy are the common methods in treatment of CRC. Despite the progress made in diagnosis and therapy, patients with CRC often develop recurrence and metastasis, which decrease the 5-year survival rate dramatically 1. Therefore, it is urgent to reveal and understand the molecular mechanisms of CRC development and to find the potential biomarkers to improve CRC diagnosis, treatment, and prognosis.

The integrin (ITG) molecules are principal cell surface receptors responsible for cell extracellular matrix interactions 2. They are formed by non-covalent associations of α and β dimers and modulate different signal transduction cascades. Up to now, 18 α and 8 β subunits have been found and form a number of distinct integrins 3. ITGs are found to be involved in regulation of a variety of cell signaling pathways including migration, invasion, differentiation, proliferation, and survival, and are associated with cancer development 4-6. Among them, integrin β4 (ITGB4), forming dimer with integrin α6 (ITGA6), is wildly studied in carcinomas 2, 7. During carcinoma progression, the integrin α6β4 is released from hemidesmosomes, which allows it to be associated with the actin cytoskeleton 8. Here, it activates RhoA, leading to membrane ruffling, lamellae formation, and the generation of traction forces and then promotes invasive and metastatic behavior 9, 10. ITGB4 is aberrantly expressed in several cancers including breast, colorectal, and lung cancers and may be positively associated with poor prognosis 7, 11, 12. However, the reports on the associations of ITGB4 with clinicopathological features and/or outcomes in colon cancer are rare.

In the present study, we utilized the large public datasets from NCBI GEO and TCGA databases and collected clinical samples in our center to investigate the expressions of ITGB4 at transcriptional level and explore its clinical significance in colon cancer. In addition, ITGB4 co-expressed genes were annotated to investigate how ITGB4 promoted colon cancer development. The putative transcription factors and microRNAs targeting ITGB4 gene and its promoter methylation were also explored to investigate how ITGB4 transcription was regulated.

## Materials and Methods

### Tumor samples

The fresh primary tumor specimens and their corresponding adjacent non-tumor tissues were obtained from colon cancer patients underwent surgery in our center from 2016 to 2017. No patients had received radiotherapy and/or chemotherapy prior to surgery. The specimens were stored at -80°C immediately after surgery. Written informed consent was obtained from all patients and all procedures were approved by the Institutional Research Ethics Committee.

### Data mining

To compare the transcriptional expression difference of ITGB4 between colon cancer tissue and adjacent tissues and to investigate the associations of ITGB4 with clinicopathological features and prognosis in patients with colon cancer, gene expression profile data with corresponding clinical data was searched and downloaded from the publicly available NCBI GEO and TCGA databases. After reviewing the searching results, GSE17536 13, 14, GSE39582 15, GSE41258 16, and GSE72970 17 datasets were finally chose because they were consisted of large number subjects (more than 50 cases), analyzed by Affymetrix microarrays, and contained overall survival information. At the same time, TCGA colon cancer dataset was also downloaded. ITGB4 was split into high expression group and low expression group by optimal cut-off values which were obtained from with X-title software based on the overall survival 18. Expressions of ITGB4 between tumor tissues and adjacent tissues were compared with paired or unpaired Mann Whitney U test, or one-way analysis of variance (ANOVA) followed by Bonferroni's multiple comparison tests. When comparing ITGB4 expression in healthy, adjacent normal, and tumor colon cancer tissues, the dataset GSE44076 was also included 19. Associations of ITGB4 with clinical features including age, gender, T stage, N stage, M stage, TNM stages, microsatellite instability (MSI) status were determined by Fisher's exact test or Chi-square test. Furthermore, Kaplan-Meier plot curves and univariate/multivariate Cox proportional hazard regression analyses were performed to explore the correlations of ITGB4 with overall survival in colon cancer. All the datasets were also integrated to investigate the clinical significance of ITGB4 in colon cancer. In addition, to investigate the related genes during ITGB4 alteration that might be involved in colon cancer development, GSE41258 and GSE72970 datasets were chose for deep analyses. Differently expressed genes (DEGs) between ITGB4 high and low groups were obtained. And the expression correlations of the genes with ITGB4 were examined by Pearson's correlation analysis. Then ITGB4 co-expressed genes with *P*-value less than 0.001 were selected and overlapped with the DEGs. The final obtained genes were annotated by the Metascape online database (http://metascape.org)^20^.

### Quantitative real-time polymerase chain reaction (qRT-PCR)

RNA extraction from colon cancer or adjacent tissue samples were performed using RiboZol reagent (Amresco, Solon, OH, USA) using the manufacturer's instructions. The concentration and purity of isolated RNA were estimated using the ND-1000 microspectrophotometer (Thermo Fisher Scientific, Waltham, MA, USA). Complementary DNA primers specific for ITGB4 amplification were as follows: Forward, 5'- TCTCTCAGAGTGAGCTGGCAG-3'; Reverse, 5'-TTCAGCAGCTGGTACTCCAC-3'. Quantitative PCR monitored with Brilliant II SYBR QPCR Low ROX Master Mix (Agilent) was performed on a MX3000P Real-Time System (Stratagene, Mississauga, ON, Canada). Relative mRNA levels of each gene were normalized to GAPDH expression. All experiments were performed in triplicate and repeated three times.

### Statistical analysis

Fisher's exact test, Chi-square test, Mann Whitney U test, and ANOVA were conducted via GraphPad Prism 6.0 software (La Jolla, CA, USA). Univariate and multivariate Cox proportional hazard regression were performed using SPSS 19.0 software. All tests were two-sided. *P* < 0.05 was considered to be statistically significant. All graphs were made using GraphPad Prism 6.0 software.

## Results

### ITGB4 was upregulated in colon cancers

**Fig 1:**
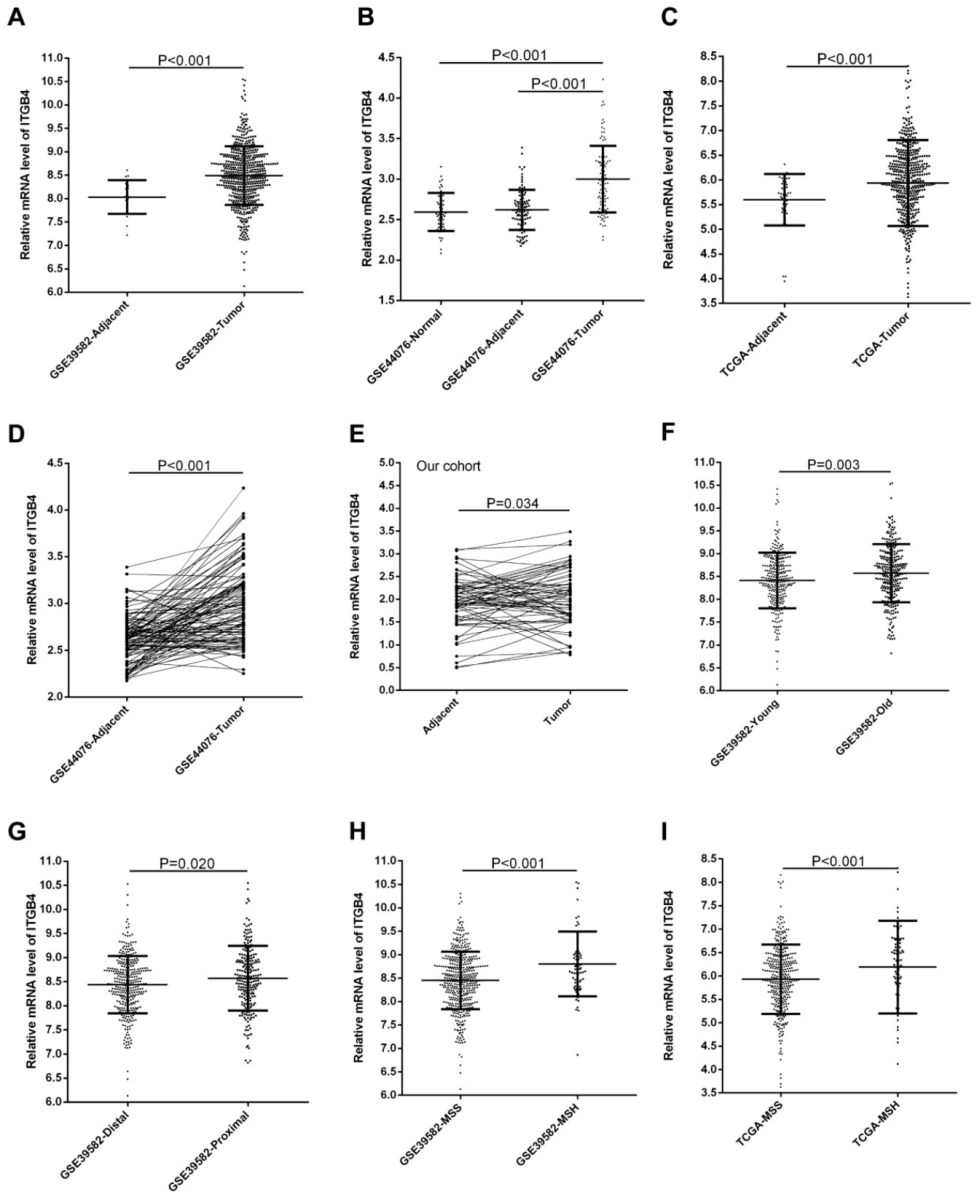
Comparison of ITGB4 expression in colon cancer tissues and adjacent tissues or in colon cancer patients with different clinical features. ITGB4 was highly expressed in colon cancer compared to adjacent tissues in GSE39582 (A, D), GSE44076 (B), TCGA cohort (C), and our cohort (D). ITGB4 was also found to be highly expressed in patients with elder onset age in GSE39582 (F), with proximal cancers in GSE39582 (G), and with MSH status in GSE39582 (H) and TCGA cohort (I). Abbreviations: TCGA, the Cancer Genome Atlas

GSE39582, GSE44076, and TCGA-colon cancer datasets contained the adjacent non-tumor tissues controls and GSE44076 also contained the data from healthy subjects. Unpaired and paired Mann Whitney U test and ANOVA were performed to compare the expressions of ITGB4 in colon cancer tissues with adjacent non-tumor tissues or tissues from healthy subjects. The statistical analyses suggested that ITGB4 mRNA expressions were up-regulated significantly in colon cancer compared with adjacent controls in GSE39582 (Figure 1A), GSE44076 (Figure 1B and D), and TCGA-colon cancer (Figure 1C), and with healthy controls in GSE44076 (Figure 1B). In addition, we collected tumor tissues and corresponding adjacent non-tumor tissues from 65 patients with colon cancer and analyzed the ITGB4 expression with qRT-PCR and also found that ITGB4 expression in tumor tissues was higher than adjacent tissues (Figure 1E).

### Association of ITGB4 with clinical features and MSI status in colon cancer

The associations of ITGB4 expression with clinicopathological parameters including gender, age, T stage, N stage, M stage, TNM stage, tumor location, and MSI status were investigated in all available datasets and in integrated analyses. We found that high ITGB4 expression was associated with elder onset age (Figure 1F), proximal tumor location (Figure 1G), and MSH status (Figure 1H) in GSE39582, with M stage in GSE41258, and with MSH status in TCGA colon cancer dataset (Figure 1I) (Table S1). In integrated analyses, high ITGB4 expression was associated with elder onset age, proximal tumor location, and MSH status (Table 1).

### Associations of ITGB4 with overall survival in colon cancer

**Fig 2:**
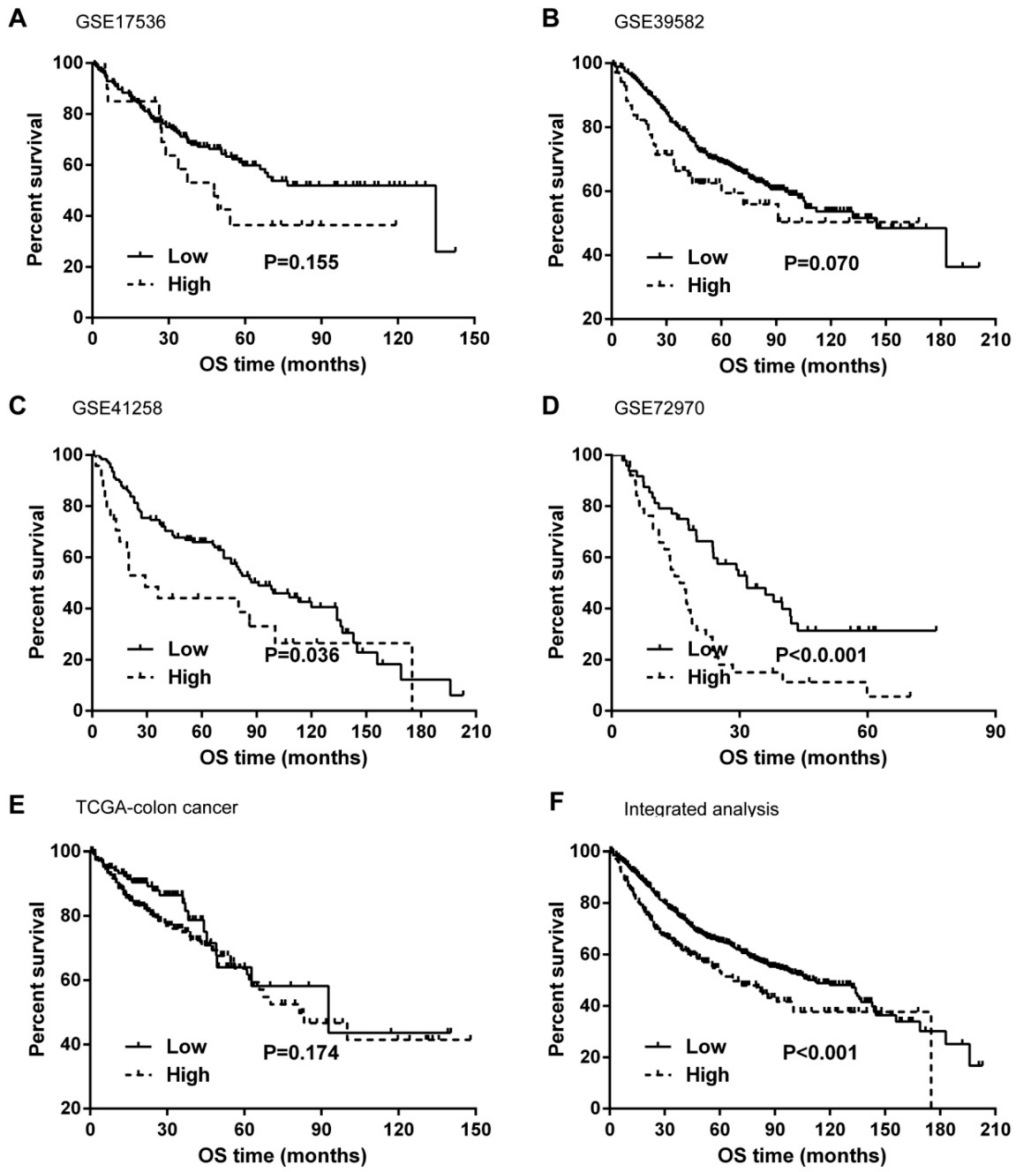
Effects of ITGB4 expression on overall survival in colon cancer analyzed by Kaplan-Meier curves. (A) dataset GSE17536; (B) GSE39582; (C) GSE41258; (D) GSE72970; (E) TCGA-colon cancer; (F) integrated analysis. Abbreviations: TCGA, the Cancer Genome Atlas

**Fig 3:**
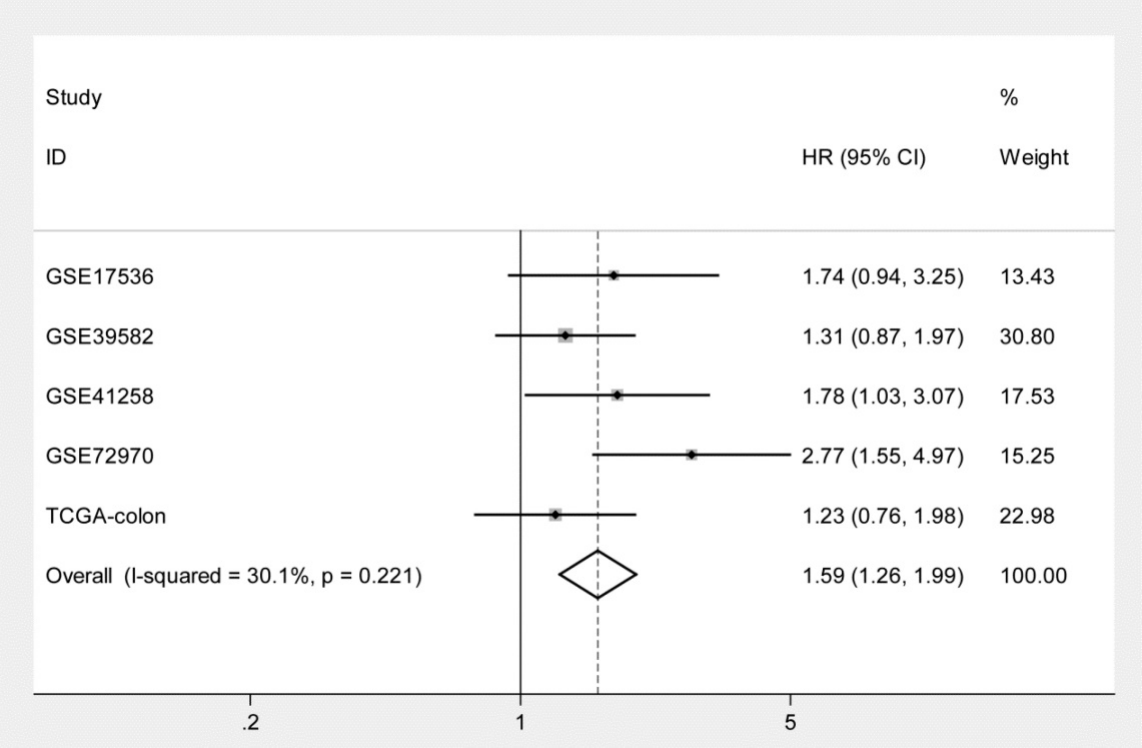
Pooling analysis of the adjusted hazard ratios for the associations of ITGB4 expression with overall survival in colon cancers from individual datasets.

Kaplan-Meier curves and univariate Cox proportional hazard regression analyses suggested that high ITGB4 expression was significantly associated with poor overall survival in datasets GSE41258 (HR=1.845, 95%CI=1.064-3.200, P=0.029; Figure 2C and Table 2) and GSE72970 (HR=2.412, 95%CI=1.459-3.988, P=0.001; Figure 2D and Table 2). A trend of association was observed in GSE39582 (HR=1.425, 95%CI=0.947-2.142, P=0.089; Figure 2B and Table 2). After adjusting the confounding factors, significant association of high ITGB4 expression with poor overall survival was found in datasets GSE72970 (adjusted HR=2.773, 95%CI=1.546-4.972, P=0.001; Table 2) and GSE41258 (adjusted HR=1.780, 95%CI=1.032-3.068, P=0.038; Table 2). Then all the datasets were integrated and high ITGB4 expression was found to be significantly associated with unfavorable overall survival in colon cancer (HR=1.574, 95%CI=1.305-1.900, P<0.001; Figure 2F and Table 2). And significant association was also found after adjusting confounding factors (adjusted HR=1.529, 95%CI=1.266-1.846, P<0.001; Table 2). In addition, the separate adjusted HRs were pooled by meta-analysis method and a significant association was found (pooled HR=1.586, 95%CI=1.262-1.992, P<0.001, fixed effects model; Figure 3). Due to the different follow-up duration in different datasets, we also analyzed the value of ITGB4 expression in predicting three years overall survival, five years overall survival, and 10 years overall survival. Significant associations of high ITGB4 expressions with 3-years OS, 5-years OS, and 10-years OS were observed in both univariate and multivariate Cox proportional regression analyses (adjusted HR3yrs=1.781, 95%CI=1.431-2.217, P<0.001; adjusted HR5yrs=1.611, 95%CI=1.318-1.968, P<0.001; adjusted HR10yrs=1.552, 95%CI=1.283-1.877, P<0.001; Table 3).

### Analyses of the genes involved in colon cancer during ITGB4 alteration

**Fig 4:**
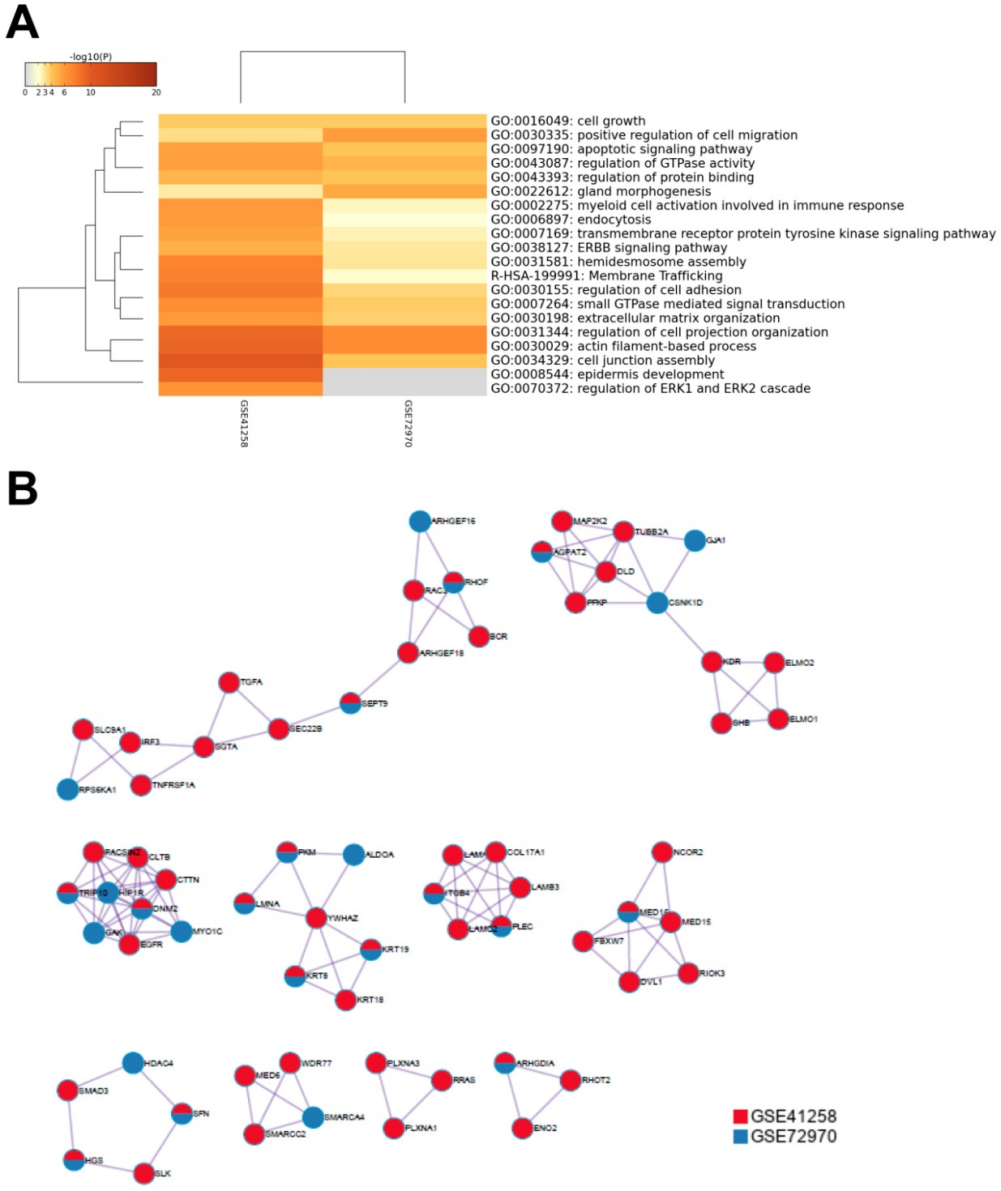
Annotations of the genes that were differently expressed in ITGB4 high and low groups and associated with ITGB4 expressions in datasets GSE41258 and GSE72970. (A) Gene ontology (GO) annotation; (B) Protein-protein interaction (PPI) network.

**Fig 5:**
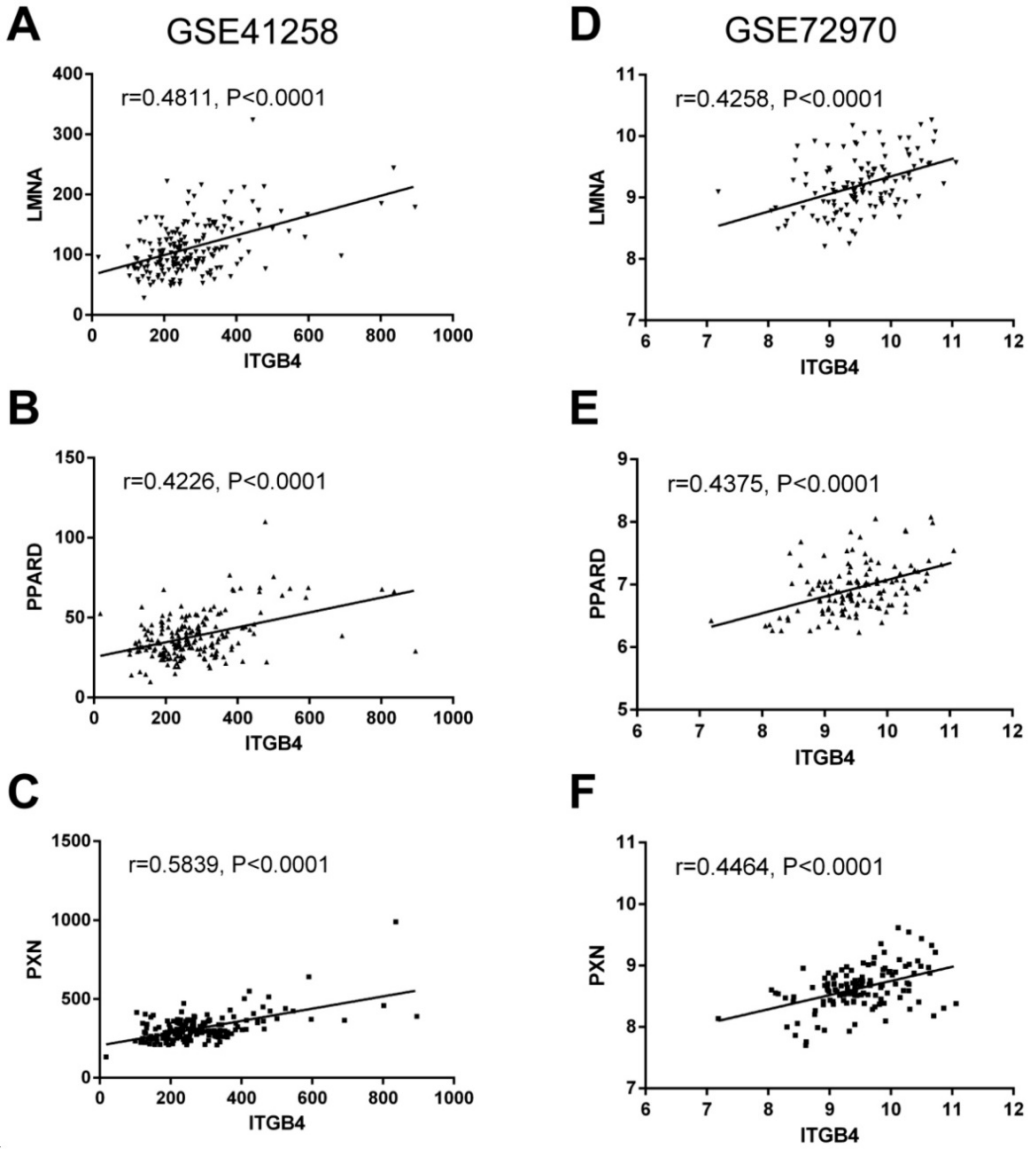
Correlations of ITGB4 expression with LMNA, PPARD, PXN, and SFN expressions in datasets GSE41258 and GSE72970. The genes mRNA expression data from datasets GSE41258 and GSE72970 was downloaded and the associations of ITGB4 with LMNA (A, D), PPARD (B, E), and PXN (C, F) were evaluated by Pearson's correlation analysis. Abbreviations: LMNA, Lamin A; PPARD, Peroxisome proliferator-activated receptor-δ; PXN, Paxillin.

To further understand the potential mechanisms of ITGB4 in colon cancer development, the genes that were differently expressed in different ITGB4 expression groups and were co-expressed with ITGB4 expression in GSE41258 and GSE72970 datasets were annotated with the Metascape online tool (http://metascape.org). A series of overlapped enriched biological processes were found in both GSE41258 and GSE72970, such as cell growth, positive regulation of cell migration, and apoptotic signaling pathways (Figure 4A). Protein-protein interaction network of the obtained genes was also constructed (Figure 4B). Then we also took several obtained genes as examples to present their associations with ITB4 expression in GSE41258 and GSE72970 datasets. The representative genes included Lamin A (LMNA) (Figure 5A and D), Peroxisome proliferator-activated receptor-δ (PPARD) (Figure 5B and E), and Paxillin (PXN) (Figure 5C and F).

### Regulation mechanism of ITGB4 ectopic expression in colon cancer

**Fig 6:**
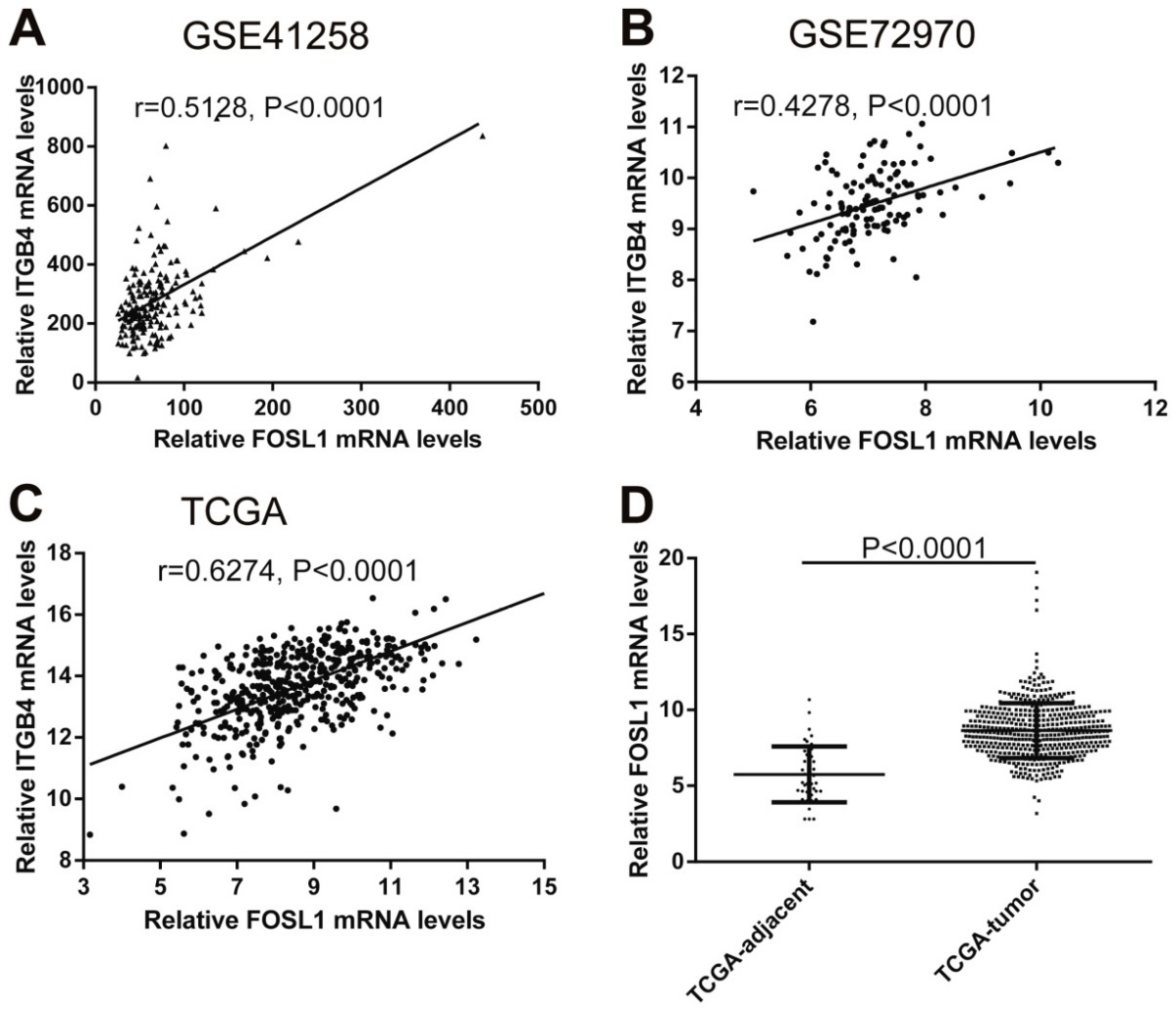
Correlations of ITGB4 expression with transcription factor FOSL1 in datasets GSE41258, GSE72970, and TCGA-colon cancer. The gene mRNA expression data from datasets GSE41258, GSE72970, and TCGA-colon cancer was downloaded and the associations of ITGB4 with FOSL1 in GSE41258 (A), GSE72970 (B), and TCGA-colon cancer (C) were explored by Pearson's correlation analysis. FOSL1 expression in tumor and adjacent tissues in TCGA-colon cancer cohort was also compared (D). Abbreviations: TCGA, the Cancer Genome Atlas; FOSL1, FOS like 1, AP-1 Transcription Factor Subunit

**Fig 7:**
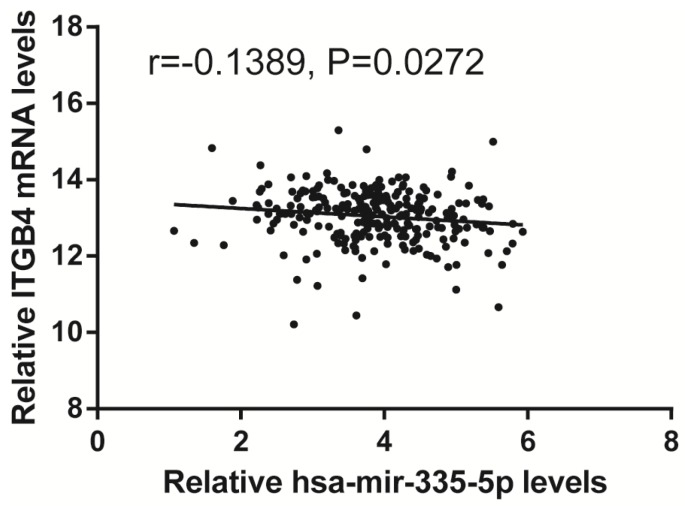
Correlation of ITGB4 expression with Hsa-mir-335-5p levels. ITGB4 mRNA and Has-mir-335-5p levels were obtained from TCGA-colon cancer cohort and explored by Pearson's correlation analysis. Abbreviations: TCGA, the Cancer Genome Atlas

**Fig 8:**
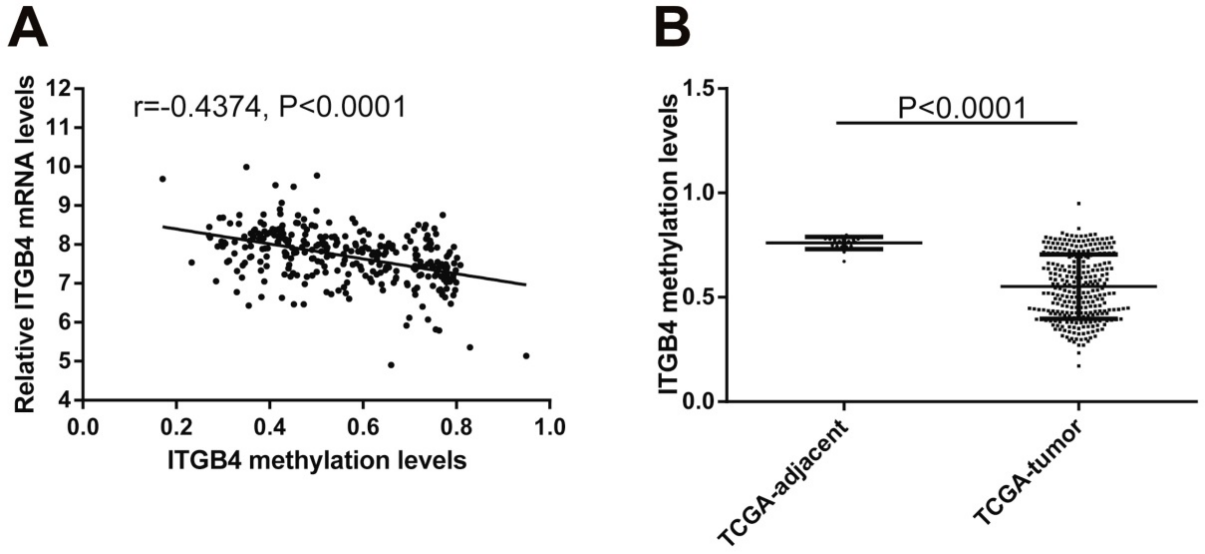
Correlation of ITGB4 expression with its methylation. ITGB4 mRNA levels and its promoter methylation levels were obtained from TCGA-colon cancer cohort and explored by Pearson's correlation analysis (A). ITGB4 promoter methylation levels in tumor and adjacent tissues were also compared (B). Abbreviations: TCGA, the Cancer Genome Atlas

Previous published works have revealed the interactions of ITGB4 promoter with some specific transcription factors (TFs) under physiologic and pathologic conditions including KLF4, RUNX1, ZEB1, TP73, GLI1, JUN, MYC, and ZKSCAN3 11, 21-27, suggesting ITGB4 transcription may be regulated by upstream transcription factors. We merged these TFs and the putative TFs binding to ITGB4 gene that were obtained from GeneHancer, a database of genome-wide enhancer-to-gene and promoter-to-gene associations and embedded in GeneCards 28. Then all the TFs were overlapped with ITGB4 co-expressed genes in GSE41258 and GSE72970 datasets. Finally, only FOSL1 (FOS like 1, AP-1 Transcription Factor Subunit) was identified, which was significantly and positively associated with ITGB4 mRNA expressions (Figure 6A and B). The association was also verified in TCGA colon cancer cohort (Figure 6C). FOSL1 was also found to be highly expressed in colon cancer tissues as well as ITGB4 in TCGA-colon cancer cohort (Figure 6D). Furthermore, we performed an *in silico* analysis using JASPAR 2018 database 29 and identified a FOSL1::JUN binding site at -48 bp to -36 bp upstream of the transcriptional start site (TSS) of ITGB4 gene. ITGB4 transcription might be also regulated by microRNAs and its promoter methylation. Ferraro *et al.* have suggested that ITGB4 was a target of miR-21 and regulated by miR-21 in colon cancer 30. With the data from TCGA-colon cancer cohort, however, we did not find the association of ITGB4 expression with miR-21 levels (data not shown). Then we predicted the microRNAs that targeted ITGB4 with comprehensive experimentally validated miRNA-gene interaction data collected from TarBase 31 and miRTarBase 32, and six microRNAs were identified including miR-1-1, miR-16-5p, miR-30a-5p, miR-1-3p, miR-155-5p, and miR-355-5p. Further analysis revealed that only miR-355-5p was slightly and negatively associated with ITGB4 expression (Figure 7). In addition, we determined the methylation of ITGB4 promoter and found methylation of ITGB4 promoter was negatively correlated with ITGB4 expression (Figure 8A) and down-regulated in colon cancer (Figure 8B). The above resulted suggested that ITGB4 might be up-regulated by the transcription factor FOSL1 and its promoter hypomethylation in colon cancer, which needed to be was needed validated in future work.

## Discussion

Our study combined multiple centers' data containing more than 1400 cases and concluded that ITGB4 was up-regulated at transcriptional levels in colon cancer tissues compared with adjacent non-tumor tissues or healthy tissues as well as in previous report 11, 33. When evaluating the association of ITGB4 with clinical features and MSI status in colon cancer, we found that high ITGB4 expression was associated with elder onset age, proximal tumor location, and MSH status. And we did not found the association of ITGB4 expression with tumor stage, which has been reported in Tai *et al*.'s study 34. The discrepancy might be resulted from the limited samples in Tai *et al*.'s study 34. Sordat *et al*. have described a particular expression pattern for ITGβ4 in CRC, which was maintained in well-differentiated carcinomas and decreased in moderately and poorly differentiated carcinomas 35. In our study, we did not assess the association of ITGB4 expression with colon cancer differentiation due to the lack of related clinical information. Importantly, we found that high ITGB4 expression was significantly associated with unfavorable overall survival in colon cancer (HR=1.574, 95%CI=1.305-1.900, P<0.001), which has not been reported previously. As ITGB4 functions by forming dimer with ITGA6, we also investigated the effects of ITGA6 expression on prognostic value of ITGB4 expression with the data from datasets GSE41258 and GSE72970 and found ITGA6 expression did not influence the association of ITGB4 expression with prognosis in colon cancer (data not shown).

Currently, little was known on the molecular mechanism of ITGB4 in colon cancer. Tai *et al.* suggested that ITGB4 together focal adhesion kinase contributes to the tumor development of colon cancer 34. Ferraro *et al.* suggested that ITGB4 is a target of miR-21 and regulates colon cancer cell migration 30. To further understand the potential mechanisms of ITGB4 in colon cancer development, we annotated ITGB4 related genes and found several biological processes such as cell growth, positive regulation of cell migration, and apoptotic signaling pathways might be involved in ITGB4 regulation of colon cancer progression. LMNA, PPARD, and PXN were the representative ITGB4 related genes and participated in regulation of cell growth, migration, and/or apoptosis. In CRC, high LMNA expression can increase tumor invasiveness and a more stem cell-like phenotype and is associated poor prognosis 36; high PPARD expression can promote tumor angiogenesis by enhancing epithelial-mesenchymal transition, migration, and invasion and is associated with significantly reduced metastasis-free survival 37, 38; and high PXN expression can promote tumor invasion and confere 5-fluorouracil resistance via ERK-mediated stabilization of Bcl-2 protein, resulting in poor patient outcomes 39, 40.

Potential regulation mechanisms of ITGB4 aberrant expression in colon cancer were also not very clear. The transcription factors, C-MYC and ZKSCAN3 have been reported to directly bind to ITGB4 promoter and regulate ITGB4 transcription in colorectal cancer 11, 27. And the interaction of ITGB4 promoter with some transcription factors in other disease is also reported, such as KLF4 in glioma 21, RUNX1 in myeloid leukemia 22, ZEB1 in prostate cancer 23, TP73 in lung cancer 24, GLI1 in ovarian cancer 25, and JUN in pancreatic cancer 26. Ferraro *et al*. have suggested that ITGB4 was a target of miR-21 and regulated by miR-21 in colon cancer 30. However, we did not found any evidence supporting these factors in regulation of ITGB4 transcription after analyzing the data from datasets GSE41258 and GSE72970. Whereas, we found that ITGB4 ectopic expression might be regulated by the up-regulation of the transcription factor FOSL1, the down-regulation of miR-335-5p, and its promoter hypomethylation in colon cancer.

In conclusion, ITGB4 is highly expressed in human colon cancer and associated with poor overall survival. The annotation of ITGB4 related genes suggested ITGB4 might promote tumor development via regulating a series of cellular pathways including cell proliferation. The elevated ITGB4 transcription in colon cancer might due to its upstream transcription factor FOSL1 up-regulation and its promoter hypomethylation. ITGB4 might be a therapeutic target and prognosis marker for individual therapy of colon cancer.

## Supplementary Material

Supplementary table.

## Figures and Tables

**Table 1:** Associations of ITGB4 expression with clinicopathological features of patients with colon cancer in the integrated analysis

Parameters	ITGB expression		P-value
	Low	High	
**Gender**			
Female	342	305	0.462
Male	421	347	
**Age**			
Young	394	289	**0.006**
Old	369	363	
**T stage**			
T0-2	97	78	0.662
T3-4	588	440	
**N stage**			
N0	371	277	0.813
N1-3	314	241	
**M stage**			
M0	527	377	0.898
M1	100	70	
**Stage**			
I-II	369	322	0.576
III-IV	345	283	
**Tumor Location**			
Distal	414	237	**< 0.001**
Proximal	266	265	
**MSI status**			
MSH	81	101	**0.001**
MSL/MSS	517	382	

Bold, P<0.05 demonstrated by Chi-square test. Abbreviations: TCGA, the Cancer Genome Atlas; MSH, microsatellite instability-high; MSL, microsatellite instability-low; MSH, microsatellite instability-stable.

**Table 2:** Associations of ITGB4 expression with overall survival in colon cancer

Datasets	Parameters	Univariate analysis					Multivariate analysis				
		HR	95%CI		P-value		HR	95%CI		P-Value	Adjusted factors
GSE17536	ITGB4 (High/low)	1.562	0.840	2.905	0.159		1.742	0.935	3.246	0.081	Grade and stage
	Grade (3/1-2)	2.191	1.254	3.826	**0.006**		1.641	0.933	2.886	0.086	
	Stage (III-IV/I-II)	4.220	2.388	7.460	**<0.001**		4.062	2.280	7.239	**<0.001**	
**GSE39582**	ITGB4 (High/low)	1.425	0.947	2.142	0.089		1.306	0.866	1.970	0.203	Age and stage
	Age (Old/young)	0.584	0.437	0.781	**<0.001**		1.800	1.341	2.416	**<0.001**	
	Stage (III-IV/I-II)	1.763	1.323	2.349	**<0.001**		1.893	1.418	2.528	**<0.001**	
**GSE41258**	ITGB4 (High/low)	1.845	1.064	3.200	**0.029**		1.780	1.032	3.068	**0.038**	Gender and stage
	Gender (Male/Female)	1.767	1.133	2.757	**0.012**		3.601	2.195	5.909	**<0.001**	
	Stage (III-IV/I-II)	3.649	2.228	5.975	**<0.001**		1.844	1.175	2.893	**0.008**	
**GSE72970**	ITGB4 (High/low)	2.412	1.459	3.988	**0.001**		2.773	1.546	4.972	**0.001**	T stage
	T (T4/T3)	1.957	1.125	3.405	**0.017**		2.494	1.396	4.454	**0.002**	
**TCGA-Colon**	ITGB4 (High/low)	1.459	0.913	2.331	0.115		1.231	0.765	1.981	0.392	Age and stage
	Age (Old/young)	1.965	1.272	3.036	**0.002**		2.454	1.559	3.863	**<0.001**	
	Stage (III-IV/I-II)	2.812	1.841	4.295	**<0.001**		3.204	2.082	4.929	**<0.001**	
**Integrated**	ITGB4 (High/low)	1.574	1.305	1.900	**<0.001**		1.529	1.266	1.846	**<0.001**	Age and gender
	Age (Old/young)	1.419	1.191	1.692	**<0.001**		1.386	1.162	1.654	**<0.001**	
	Gender (Male/Female)	1.221	1.025	1.456	**0.025**		1.250	1.049	1.491	**0.013**	

Bold, P<0.05 demonstrated by Cox proportional hazard regression analysis. Abbreviations: TCGA, the Cancer Genome Atlas; HR, hazard ratio; CI, confidence interval.

**Table 3:** Associations of ITGB4 with 3 years, 5 years, and 10 years-overall survival in colon cancer

Parameters		Multivariate analysis			
		HR	95%CI		P-value
3yrs OS					
ITGB4 (High/low)		1.781	1.431	2.217	**<0.001**
Age (Old/young)		1.114	0.900	1.380	0.320
Gender (Male/Female)		1.150	0.928	1.426	0.202
5yrs OS					
ITGB4 (High/low)		1.611	1.318	1.968	**<0.001**
Age (Old/young)		1.245	1.029	1.507	**0.024**
Gender (Male/Female)		1.205	0.995	1.459	0.056
10yrs OS					
ITGB4 (High/low)		1.552	1.283	1.877	**<0.001**
Age (Old/young)		1.335	1.117	1.596	**0.002**
Gender (Male/Female)		1.251	1.047	1.496	**0.014**

Bold, P<0.05 demonstrated by Cox proportional hazard regression analysis. Abbreviations: HR, hazard ratio; CI, confidence interval; yrs, years.
