# Supplementary material for: ITGB4 is a novel prognostic factor in colon cancer
Source: J Cancer. 2019 Aug 28;10(21):5223–33. doi: 10.7150/jca.29269 (PMC6775604; doi:10.7150/jca.29269)
Supplement: Supplementary file 1 — Supplementary table. [file jcav10p5223s1.pdf]

Supplementary information

Table S1. Associations of ITGB4 expression with clinicopathological features of patients with colon cancer in individual cohorts

Parameters	GSE17536			GSE39582			GSE41258			GSE72970			TCGA-Colon		
	Low	High	P-value	Low	High	P-value	Low	High	P-value	Low	High	P-value	Low	High	P-value
**Gender**															
Female	23	58	0.155	180	76	0.610	60	12	0.892	19	17	0.631	60	142	0.699
Male	37	59		224	86		66	14		29	21		65	167	
**Age**															
Young	33	50	0.122	213	70	**0.041**	65	11	0.389	25	18	0.664	58	140	0.836
Old	27	67		191	92		61	15		23	20		67	169	
**T stage**															
T0-2				46	14	0.409	23	5	0.907	1	2	0.577	27	57	0.452
T3-4				348	138		103	21		39	29		98	252	
**N stage**															
N0				221	81	0.555	68	11	0.279	6	4	1.000	76	181	0.669
N1-3				173	71		58	15		34	27		49	128	
**M stage**								0.06							
M0				343	139	0.132	91	14	0.065				93	224	0.574
M1				49	12		35	12					16	46	
**Stage**															
I-II	22	59	0.082	217	84	0.688	56	10	0.575				74	169	0.527
III-IV	38	58		187	78		70	16					50	131	
**Tumor Location**															
Distal				259	83	**0.005**	65	9	0.097	32	18	0.072	58	127	0.443

Proximal	145	79		47	14		15	19		59	153	
**MSI status**												
MSH	45	30	**0.034**	23	7	0.268				13	64	**0.018**
MSL/MSS	320	124		92	16					105	242	

Bold, P<0.05 demonstrated by Chi-square test.

Abbreviations: TCGA, the Cancer Genome Atlas; MSH, microsatellite instability-high; MSL, microsatellite instability-low; MSH, microsatellite instability-stable.
